# Tooth Eruption Results from Bone Remodelling Driven by Bite Forces Sensed by Soft Tissue Dental Follicles: A Finite Element Analysis

**DOI:** 10.1371/journal.pone.0058803

**Published:** 2013-03-15

**Authors:** Babak Sarrafpour, Michael Swain, Qing Li, Hans Zoellner

**Affiliations:** 1 The Cellular and Molecular Pathology Research Unit, Department of Oral Pathology and Oral Medicine, Faculty of Dentistry, The University of Sydney, Westmead Hospital, Westmead, New South Wales, Australia; 2 Biomaterials Science Unit, Faculty of Dentistry, The University of Sydney, Sydney, New South Wales, Australia; 3 School of Aerospace, Mechanical and Mechatronic Engineering, Faculty of Engineering, The University of Sydney, Sydney, New South Wales, Australia; Glasgow University, United Kingdom

## Abstract

Intermittent tongue, lip and cheek forces influence precise tooth position, so we here examine the possibility that tissue remodelling driven by functional bite-force-induced jaw-strain accounts for tooth eruption. Notably, although a separate true ‘eruptive force’ is widely assumed, there is little direct evidence for such a force. We constructed a three dimensional finite element model from axial computerized tomography of an 8 year old child mandible containing 12 erupted and 8 unerupted teeth. Tissues modelled included: cortical bone, cancellous bone, soft tissue dental follicle, periodontal ligament, enamel, dentine, pulp and articular cartilage. Strain and hydrostatic stress during incisive and unilateral molar bite force were modelled, with force applied via medial and lateral pterygoid, temporalis, masseter and digastric muscles. Strain was maximal in the soft tissue follicle as opposed to surrounding bone, consistent with follicle as an effective mechanosensor. Initial numerical analysis of dental follicle soft tissue overlying crowns and beneath the roots of unerupted teeth was of volume and hydrostatic stress. To numerically evaluate biological significance of differing hydrostatic stress levels normalized for variable finite element volume, ‘biological response units’ in Nmm were defined and calculated by multiplication of hydrostatic stress and volume for each finite element. Graphical representations revealed similar overall responses for individual teeth regardless if incisive or right molar bite force was studied. There was general compression in the soft tissues over crowns of most unerupted teeth, and general tension in the soft tissues beneath roots. Not conforming to this pattern were the unerupted second molars, which do not erupt at this developmental stage. Data support a new hypothesis for tooth eruption, in which the follicular soft tissues detect bite-force-induced bone-strain, and direct bone remodelling at the inner surface of the surrounding bony crypt, with the effect of enabling tooth eruption into the mouth.

## Introduction

### The Development and Eruption of Teeth

#### Developing teeth are surrounded by soft tissue follicle encased in a bony crypt

**Figure 1:**
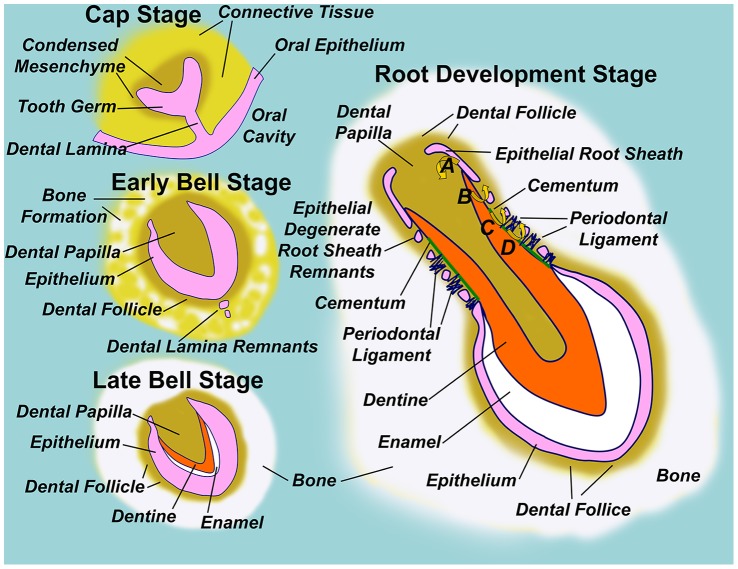
Diagram illustrating normal tooth formation. In the early embryo, the oral cavity is separated from underlying connective tissues by a stratified squamous epithelium. A ridge of epithelium invades connective tissue to form the dental lamina, while individual tooth germs are seen at the ‘Cap Stage’ of tooth development as ‘Dome-shaped’ thickenings in the dental lamina surrounded by condensed mesenchyme. Degeneration of the dental lamina to epithelial remnants isolates the tooth germ from the oral epithelium in the ‘Early Bell Stage’, so named because tooth germ epithelium remodels to a bell-like form, such that the inner surface defines the shape of the tooth crown and encloses condensed mesenchyme of the dental papilla, which is the future dental pulp. The dental follicle comprises condensed mesenchyme immediately surrounding the ‘bell’ epithelium, and bone begins to form surrounding the follicle. In the ‘Late Bell Stage’ of tooth development, inductive signals from the epithelium drive differentiation of dentine forming odontoblasts in the adjacent dental papilla. Progressive layers of dentine encroach on the dental papilla space, while dentine itself acts as a further inductive signal driving inner epithelial cells of the tooth germ to differentiate to enamel forming ameloblasts. Analogous to dentine, layers of enamel are deposited by ameloblasts at the expense of tooth germ epithelium, so that the tooth crown has formed by conclusion of the ‘Late Bell Stage’. Throughout, bone formation continues around the dental follicle, and the tooth germ becomes enclosed within a bony crypt embedded within the jaw. Root development is initiated by downgrowth of epithelial cells at the ‘lip of the bell’ to form an epithelial root sheath. Root sheath epithelial cells instruct dentine formation in the underlying papilla (A), and respond to the newly formed dentine by degenerating into root sheath remnants. Dentine thus becomes exposed to cells of the dental follicle, which respond by cementoblast differentiation (B). Cementoblasts then layer cementum over the exposed dentine, while cementum itself acts as a further inductive signal to cells of the follicle to form the periodontal ligament which anchors cementum to the surrounding bony crypt via dense collagen fibres (D). In this way, the root sheath defines root shape with the furthest extent of root sheath downgrowth defining the root apex, and inductive steps following root sheath degeneration establish the necessary bony attachment of teeth via the periodontal ligament [1]–[3].

Although the bony anchorage of teeth provides a firm basis for biting, this does necessitate the development and subsequent eruption of teeth through bone, and we seek to understand the mechanisms controlling the necessary bone remodelling. As outlined in Figure 1, teeth form through complex interactions between epithelium and the underlying connective tissues, and before eruption are surrounded by a soft tissue dental follicle which is further encased by a bony crypt of dense cortical bone. As the tooth erupts, this bony crypt eventually becomes confluent with the jaw’s cortical bone to form the ‘lamina dura’. The periodontal ligament differentiates from that part of the soft tissue dental follicle that overlies the developing tooth, and anchors the root to the lamina dura [1]–[3].

#### The position of teeth is determined by the balance of eruptive and external forces

An eruptive force is widely invoked as driving teeth out of the jaw bones into eventual occlusion with teeth from the opposing jaw [4], [5], although the origin of such an eruptive force remains unclear. Separately, forces from the oro-facial musculature also play a major role in precisely positioning teeth within the jaws, as evidenced by the gross outward or inward displacement of teeth upon loss or gain of facial or tongue tissues, respectively [6]–[8]. Supporting the effect of such muscular forces, is the movement of teeth by routine clinical orthodontic treatment [9].

#### Current explanations for the eruptive force are inconsistent with observation

Although an eruptive force seems biologically and clinically apparent, the actual origin of such a force is unknown. While an eruptive force of 0.1–1.1 g has been measured for the continuously growing rodent incisor [10], the unusual continuous formation of root apex in these teeth may make this inappropriate for comparison with most other teeth [5], [11]. The periodontal ligament vasculature has been suggested as a possible source of the eruptive force [12], but this seems inconsistent with the separate concept that the vasculature of the periodontal ligament is adapted for rapid emptying upon occlusal loading [13].

Root elongation has also been suggested as another possible origin of the eruptive force, but this seems inconsistent with post eruptive movement following completed root formation [14], [15], while rootless teeth are able to erupt to oral cavity [16], [17]. In addition, newly formed dentine at the apex of the growing root is unmineralized and readily deformed by trauma, and while this is seen clinically in traumatic dilaceration [18], there is no sign of similar unmineralized dentine deformation in normally erupting teeth.

Formation of the periodontal ligament and collagen contraction have also been suggested to generate eruptive force [19]. However, the rate of collagen turnover is much higher than the speed of tooth eruption, while the eruption rate is unaffected by inhibitors of collagen synthesis [20]. Fibroblast contraction remains a possible source of eruptive force [19], [21], [22], but this seems inconsistent with the effect of collagen synthesis inhibitors because fibroblasts would need to both bind and remodel collagen fibres in order to mediate eruption by contraction.

In this manuscript, we consider the possibility that there is no true eruptive force. Instead, we suggest that similar to the effect of cheeks, tongue and opposing occlusion in positioning teeth within the mouth, there is remodelling of tissues in response to functional forces on the jaws such that teeth are carried upwards out of the bone and into the mouth. This requires that tissues are able to both sense and respond to functional stress. Thus an important objective of the current study was to determine which tissues are biomechanically best suited for detection of such a functional stress.

### Functional Deformation of the Jaw Bones as a Sensor Mechanism Driving Tooth Eruption

#### Bony impaction indicates that bone remodelling for tooth eruption is not pre-programmed but instead is responsive to local signals

Tooth eruption clearly involves remodelling of the bony crypt, and this is consistent with altered expression by cells in the soft tissue dental follicle of genes regulating bone resorption and formation [23]–[27].

**Figure 2:**
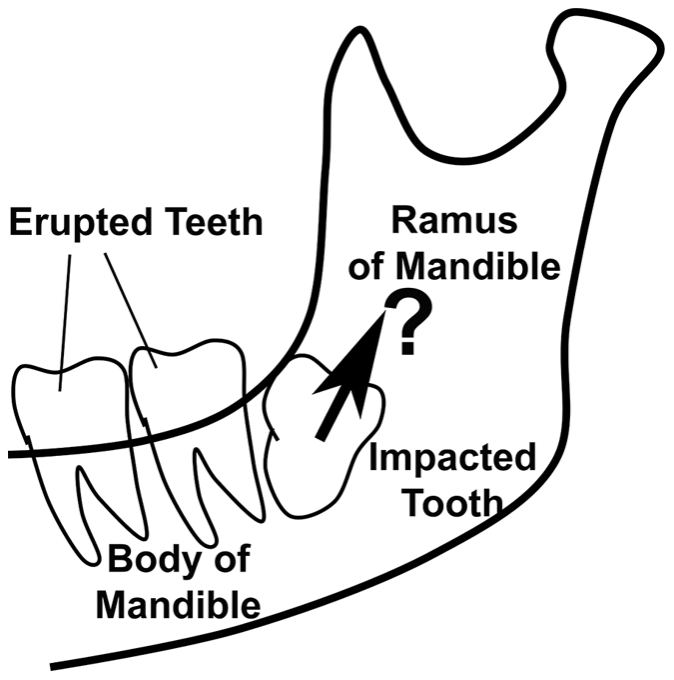
Diagram outlining the position of a mandibular third molar with bony impaction. The outline of the body and ramus of the mandible is shown, as is the location of first and second molar teeth which have erupted normally into the mouth. A third molar tooth is shown, which has formed in such a way that the crown is orientated up into the ramus of the mandible rather than into the oral cavity. Despite this being a common clinical event, there are no reported cases of third molars erupting along the path indicated by the arrow. Instead, teeth with bony impaction appear to reach a stable position, from which further eruption does not proceed. We conclude that factors other than tooth orientation determine the path of tooth eruption.

While it seems reasonable to assume expression of bone remodelling genes is pre-programmed to guarantee crown-ward eruption of teeth into the mouth, clinical evidence indicates that this is not the case. In particular, occasional teeth form in an orientation that results in failed eruption, clinically termed ‘impaction’. Although many impacted teeth seem blocked by other teeth, occasional ‘bony impactions’ occur where no clear obstruction is present (Figure 2) [28]. If the orientation of bone remodelling for tooth eruption were pre-programmed and inherent to the dental follicle and periodontal ligament, then true stable bony impaction should not occur, and instead teeth would continue to erupt till encountering a free bone surface. We conclude that bony impactions reveal a critical role for local signals regulating the directionality of bone remodelling for tooth eruption, so that identifying the nature of such local signals is important.

#### Jaw bones deform under physiological forces and this is associated with bone remodeling

The jaw is typically subject to high levels of muscular force during chewing, as well as to lower forces associated with speech, breathing and near constant postural muscle activity [7], [29]–[36]. Bone responds to functional stress by either resorption or apposition in accordance with the nature and direction of the force applied, so that bones constantly remodel to accommodate the forces they must bear [37]–[40]. It is widely accepted that masticatory function influences the morphology and development of the jaws across species [41]–[44], and it seems logical to suggest a role in tooth eruption.

#### Bone remodelling of tooth eruption may be directed by functional deformation of bone which is amenable to investigation by finite element analysis

Finite element analysis (FEA) is widely accepted as an effective non-invasive tool for studying the influence of mechanical forces on the relationship between form and function in biological systems [45]–[51]. Notably, FEA-based prediction of the heterogeneous distribution of oral bone can be correlated to radiological clinical data [52], [53]. We recently reported the results of two dimensional FEA, suggesting a role for functional strain in the continuous emergence of erupted teeth into opposing occlusion [54]. In brief, we found that the strain on the inner surface of the lamina dura was consistent with lamina dura remodelling in a way that would account for continuous eruption. Nonetheless, the recent study was limited by examining a conceptually constructed two dimensional model, rather than the events in a more anatomically realistic three dimensional model of teeth in an actual jaw. Also, our previous study did not investigate unerupted teeth, but examined only continuous eruption of a tooth already in the mouth [54].

Notwithstanding a critical role for bone as opposed to soft-tissue strain in driving eruptive bony remodelling [54], it is interesting that separate analysis by others suggests a possible role for periodontal ligament in driving continuous tooth eruption [55], [56]. From the above, it remains unclear if functional bone strain is sufficient to account for bone remodelling in tooth eruption, or if perhaps functional strain in the dental follicle and or periodontal ligament soft tissues, plays a more important role.

In this paper, we describe a three dimensional finite element (FE) model of a child mandible with multiple unerupted teeth, examining the distribution, directionality and magnitude of strain in both soft and hard tissues relevant to tooth eruption, with a view to identifying the tissue most likely to act as a sensor for functional strain and hence most likely responsible for regulating the direction and rate of tooth eruption. At the stage of development studied, six teeth comprising the mandibular canines and premolars are known to be in a phase of active eruption, while the two second molar teeth have crowns but no clear root development and are in a pre-eruptive phase [57], [58]. This provides convenient comparison within the model, of the eruptive response with pre-eruptive teeth.

#### The definition of strain and hydrostatic stress in finite element analysis

With regard to three dimensional FEA, clarity in the precise definition of strain and hydrostatic stress used in the current study is important to interpret results. ‘Strain’ relates to distortion and is expressed as a unitless measure derived from the distance between two points before (L_0_) and after (L_1_) application of a force, such that strain (*S*) = (L_1_–L_0_)/L_0_. Equivalent strain represents a scalar measure of overall deformation and is calculated from the component principal strains by the following equation,
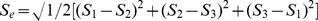
where: *S_e_* denotes the equivalent strain, *S*_1_, *S*_2_, and *S*_3_ stands for the first, second and third principal strains in the three Cartesian axes [54], [59]. Strain relates to ‘stress’, which is a measure of the internal force intensity acting within a deformable body. Stress (σ) is expressed in units of Pascals (Pa), and calculated such that σ (Pa) = Force (N)/Area (m^2^).

In three dimensional FEA, discrete volumetric finite elements are defined within a ‘mesh’, and are connected to one another through adjacent ‘nodes’ located at the border of each element. Application of force to the FEA model may lead to changes in the volume of each element, secondary to displacement of individual nodes. Whilst there may be elongation and hence ‘tensile strain’, there may also be concomitant narrowing and hence ‘compressive strain’. Hence by vectorial analysis, it is possible to quantify the strain in elements [60]. Amongst the assumptions necessary to conduct the current study, was that the tissues modelled behave primarily in an elastic and isotropic manner, so that stress relates directly to strain as per Young’s modulus and Poisson’s ratio in the analysis performed.

Compression of a bony surface adjacent to soft tissues results in resorption while bone is deposited on surfaces where there is tension [61], [62]. ‘Hydrostatic stress’ (alternatively hydrostatic strain) represents a measure to quantify the net compression or tension in each FE, by effectively averaging the stress components as per the following equation: Hydrostatic stress: 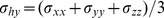, in which *σ_xx_, σ_yy_, σ_zz_* denotes the stress components along the three Cartesian axes respectively [60].

### Aims of this Study

As outlined above, we aimed to use three dimensional (3D) FEA to examine the possible role of bite forces in driving tooth eruption. This was done by first determining the tissue most likely to act as a relevant mechanosensor for bite-force induced stress and strain, and then examining relevant changes in these tissues with regard to bone remodelling for tooth eruption. In addition to constructing the necessary FEA model, we also developed a new measure in order to more meaningfully interpret hydrostatic stress changes in our model. We have termed this measure the ‘Biological Response Unit’ (BRU), which has SI units of Nmm and provides a convenient numerical quantity for interpretation of the biological significance of hydrostatic stress obtained from FEA. Through this work, the current study provides the theoretical basis for a new model of tooth eruption, in which dental follicle soft tissues detect bite-force induced jaw strain, and thus instruct bone remodelling that leads to tooth eruption.

## Materials and Methods

### Construction of a Three Dimensional (3D) Model of a Child’s Jaw

#### Acquisition of images from computerized axial tomography scan data

A cadaver specimen of a mandible from a child estimated from dental development to be eight years old was kindly selected and loaned by Dr M Robinson of the Discipline of Anatomy and Histology, The University of Sydney. This was from an extensive collection of skeletal remains obtained in the late 19th and early 20th Centuries by the University, and for which there are no available records detailing provenance. As the specimen comprised part of a teaching and research collection obtained up to a century prior to the establishment of human research ethics mechanisms, human ethics approval was not obtained, while it was also not possible to obtain informed verbal or written consent. The mandible contained eight unerupted teeth at differing stages of formation and eruption. Computerized tomography scan data were obtained with a Kodak 9500 cone beam 3D system, adjusted for scanning the whole mandible with a tube voltage of 60 kV and X-ray tube current of 15 mA at a voxel resolution of 200×200×200 µm. A total number of 445 slices in DICOM (Digital Imaging and Communications in Medicine) format was generated by the system.

**Figure 3:**
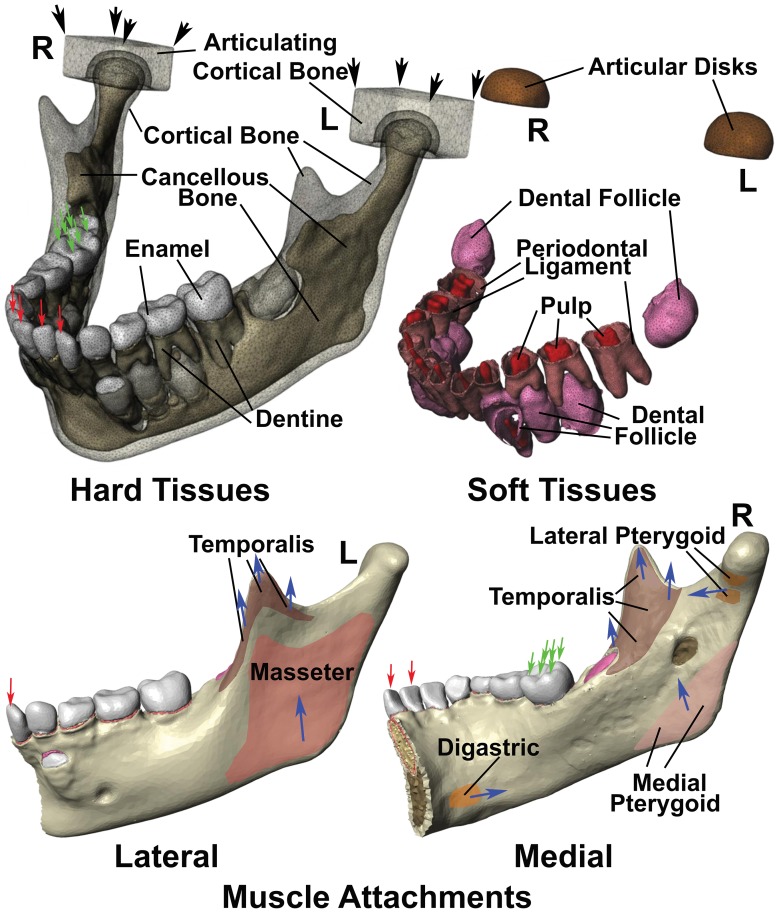
Diagrams illustrating the finite element model constructed in this study. Illustrated are the boundary conditions, as well as the muscle attachments on the lateral left and medial right surfaces of the mandible, while left (L) and right (R) joints are indicated respectively. Hard tissues modelled included cortical bone, cancellous bone, enamel and dentine. Two solid blocks with the properties of cortical bone were modelled in replacement of the base of skull. Soft tissues included dental follicle, periodontal ligaments and dental pulps, while articular disk material with the physical properties of cartilage were modelled between the mandible and articulating cortical bone blocks. The lateral surface of the mandible had only temporalis and masseter muscle attachments, while attachments for the digastric, temporalis, lateral pterygoid and medial pterygoid muscles were modelled on the medial surface. The direction of muscle force is indicated with blue arrows. The articulating cortical bone blocks were assumed fastened at the corners indicated with black arrows, while in the case of incisor biting, single fixed points were assumed at all four incisor edges (red arrows), with muscle traction generating strain within the constructed model was applied. Right molar bite force was also modelled by fixing 6 points on the outer and upper surfaces of the right first molar as indicated (green arrows), and applying muscle traction.

Scanned images were imported into an image processing program (ScanIP Simpleware, UK), which stacks the images for visualisation and segmentation based upon grey-scale density corresponding to different degrees of mineralization. Segmentation generated a volumetric model but this could not be fully automated and some manual correction was required. In this study, the cortical and cancellous bone, enamel, dentin, pulp, periodontal ligament and dental follicle were segmented individually, and corresponding masks were created by interpolation and filling of the borders to achieve accurate anatomical modelling (Figure 3). To simulate the temporomandibular joint, a 2 mm thick elastic material was modelled over condylar surfaces, while all generated masks were imported to ScanFE (Simpleware, UK) to allow volumetric meshing by using +FE free algorithm and −24 for compound coarseness.

#### Properties of the Model for Finite Element Analysis

The generated model yielded a total of 1,854,710 tetrahedral elements with 350,289 nodes, and was imported into a FEA program ABAQUS 6.9 (ABAQUS Inc, Providence, RI). Dentine, pulp, enamel, periodontal ligament, dental follicle, cortical bone and cancellous bone were assigned relevant mechanical properties as indicated in Table 1, according to values established in the literature [53], [63]–[66]. Please note that although the mechanical properties of bone do vary according to the direction of force applied, these differences are nonetheless fairly small in all three directions [67], [68] and to a considerable extent lead to very close agreement between experimental and numerical results [64], [69]. For this reason, use of linear isotropic elastic properties seems justifiable in anatomical modelling under physiological loading [70]. As such, tissues were treated as homogeneous, isotropic and linearly elastic materials.

**Table 1:** The number of finite elements, volumes, as well as the physical properties assigned to each tissue type in the finite element model used in this study.

Tissue Type	Elements	Volume mm^3^	Young’s Modulus (MPa)	Poisson’s Ratio
Cancellous Bone	240470	8854	1,500	0.30
Condylar Elastic Support - Anterior	12114	563.44	44.1	0.40
Condylar Elastic Support - Posterior	9130	363.66	0.49	0.49
Cortical Bone & Lamina Dura	640034	20660	15,000	0.30
Dental Follicle & Periodontal Ligament	189320	1971	12	0.45
Dental Pulp	47935	597.9	2	0.45
Dentine	325635	3383	18,600	0.31
Enamel	158181	2907	84,100	0.20

Young’s modulus and Poisson’s ratio for each tissue modelled was taken from the literature including for enamel, dentine, cancellous bone [53], [63], Condylar Elastic Support [66], and dental pulp [65]. Cortical bone and lamina dura were assumed to have the identical properties [53], [63], as were the periodontal ligament and dental follicle [64].

Sites for attachment of masticatory muscles were defined according to the established anatomical literature, in which the force from the following muscles was modelled: superficial and deep masseter; anterior, middle and posterior fascicles of temporalis; medial and lateral pterygoid; and the anterior belly of the digastric [71], [72] (Figure 3).

The temporomandibular joint is comparatively resilient, with the condyle impacting against a fibrous articular disk in the anterior region, and articular ligament material in the posterior region. To model this, two blocks of cortical bone were positioned on the articular surfaces of condyles, and the space in-between filled by a 2 mm thick layer of elastic material (Figure 3), while the mechanical properties assigned to the anterior and posterior parts of this area were analogous to the anterior and posterior articular joint materials as indicated in Table 1 [64], [66].

### Analysis of the Model Under the Influence of Simulated Bite Forces

#### Loading and boundary conditions

The model was studied as loaded in two different relevant physiologic settings, representing incisive and unilateral molar biting forces, respectively. Muscular anatomical insertion areas were defined by orthogonal components where XY was the horizontal transverse plane, ZY the sagittal plane and XZ the coronal plane. Directions for muscle forces were defined as per direction cosines derived from the vectors of muscular attachment [73]. The value of each masticatory force (M_ir_) in Newtons (N) was calculated from the product of the cross-sectional area of the muscle considered (X_MI_) in cm^2^, a constant for skeletal muscles (K) in N/cm^2^, and the scaled value of each muscle (EMG_MI_) contraction relative to its maximum response to the incision and unilateral molar biting, i.e. M_ir = _ [X_MI._K]⋅EMG_MI_ [73], [74].

With regard to boundary conditions for the temporomandibular joints, for both biting functions, the constraints were placed on the two bone blocks in all three axes at the upper four corner nodes. When modelling incisive bite force, the model was also restrained from vertical movement at incisive surfaces of the four incisors, while for unilateral molar biting, the restraints were placed at the buccal and central thirds of the occlusal surfaces on the right first molar [42] (Figure 3).

Since there are no consistent *in vivo* data of forces for each masticatory muscle in 8 year old children, we adjusted available adult muscle values until the force generated due to incision and molar biting came within the published range of these functions for children. In our model, the reaction forces were computed as 92.44 N and 288.72 N for incisive and molar biting forces respectively, which are consistent with the average values for incisive (∼100 N) [75], and molar forces (296.3 N) reported for school aged children [76].

#### Definition of coronal and apical dental follicle soft tissue caps surrounding unerupted teeth for numerical analysis

**Figure 4:**
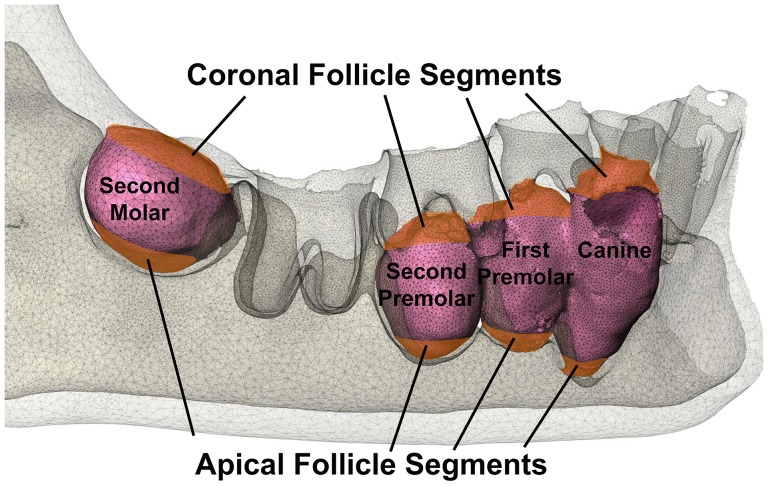
Diagram illustrating coronal and apical follicle segments of right-unerupted teeth for quantitative analysis. While only the surface of each dental follicle is shown, the entire thickness of dental follicles was examined during quantitation.

For reasons to be outlined in the results below, it became essential to perform a detailed analysis of the biomechanical responses in the dental follicle. For each unerupted tooth, two ‘cap-shaped’ volumes of soft tissues in the coronal and apical parts of the dental follicle were defined for detailed analysis, such that the boundary of each soft-tissue cap was 3 mm along the tooth’s long-axis and towards the mid-portion of the tooth from the start of the coronal or apical soft tissues respectively (Figure 4). Volume and hydrostatic stress were determined for all the elements within these soft tissue caps and these values scrutinized by three different methods to be detailed below, each of which demonstrated a different aspect of the tissue’s responses to the biting forces considered.

#### Graphical display of the relative percentage volume distribution according to hydrostatic stress in follicular soft tissue caps

In order to gain a visual impression of the data, distribution plots of the relative percentage of volume occupied by elements with hydrostatic stress values of defined ranges were prepared, considering the apical and coronal soft tissue caps of individual teeth during both incisive and right molar bite force application. Summated volumes were calculated for all FEs in defined ranges of hydrostatic stress, with rising increments of 0.005 MPa between 0.005 MPa and 0.07 MPa, while FE volumes above and below the maximum and minimum hydrostatic stress values indicated were summated accordingly. The relative percentage volume for each of these hydrostatic stress ranges was then calculated, and plotted against absolute values for hydrostatic stress, so that direct comparison could be made of the distribution volumes subjected to defined levels of compression or tension accordingly.

Whilst some general patterns were seen in the distribution plots of individual teeth, it was recognized that local idiosyncratic responses in individual teeth could obscure any general responses sought for. To minimize this influence, the data was further pooled for apical and coronal soft tissue caps, respectively. Results for the second molars were excluded from these pooled tooth analyses, as these teeth are not actively eruptive at the stage of jaw development considered here.

#### Quantification of total relative tissue volumes under compression or tension in coronal and apical soft tissue caps of unerupted teeth

Further analysis was to evaluate the proportion of tissue volume in soft tissue caps under either tension or compression subject to bite force. This was done by summation of FE volumes under either compression or tension in each apical or coronal tissue cap. To allow for different total soft tissue volumes across reading sites, the data was normalized in line with relative percentage values. To simplify the tabular display, only relative percentage values for compression are shown, because relative percentage values for tension are readily derived by subtraction from 100% of values for compression. Larger tissue volumes were thus observed to be in compression if the relative percentage values for compression exceeded 50%, while larger volumes of tissue were seen to be in tension if relative percentage values for compression were less than 50%. This analysis was performed for individual coronal and apical soft tissue caps, as well as for coronal and soft tissue caps of pooled canines and premolars, respectively. Once again, because the second molars do not erupt at this stage of development, these teeth were excluded from the pooled analysis.

#### Evaluation of likely biological dose-responses to hydrostatic stress levels by definition of a ‘Biological Response Unit’ and use of this measure to quantify compression and tension in coronal and apical soft tissue caps

Cellular and tissue responses are usually graded, such that lower levels of stimulation result in lesser responses compared with higher levels of stimulation, whilst such dose-response effects are maintained across synergistic interactions between multiple stimuli [77]–[80]. Since our interest resides in the potential cellular responses to hydrostatic stress with regard to bone resorption or apposition, we developed an approach for evaluation of the dose-effect of hydrostatic stress in the tissues concerned. To accommodate the need to relate tissue volume to the level of hydrostatic stress, and assuming that there is a linear relationship between the biological responses and both hydrostatic stress and tissue volume, a new measure was introduced as a product of hydrostatic stress (in units of MPa) multiplied by the tissue volume (in units of mm^3^). Since 1 MPa is defined as 1,000,000 N/m^2^, which equates to 1,000,000 N/1,000,000 mm^2^, the derived measure used in the current study has units of Nmm (from 1,000,000 N mm^3^/1,000,000 mm^2^). Increasingly negative values of this derived measure are assumed to correspond to increasingly greater biological responses to compression, while increasingly positive values are assumed to correspond to increasing biological responses to tension. For this reason the derived measure was termed ‘Biological Response Unit’ (BRU) in the current study. Please note that while Nmm can also represent Energy or Moment units in physics and mechanics, we will persist with the term BRU in this manuscript directly relating to biological response in affected tissues.

**Figure 5:**
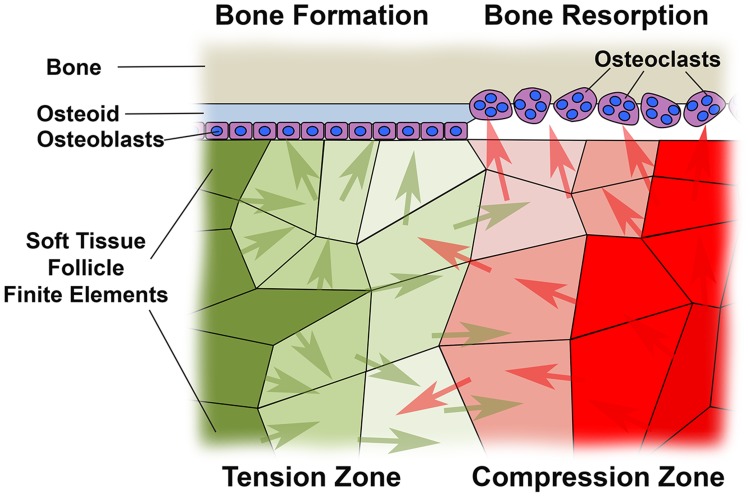
Diagram illustrating the significance of ‘Biological Response Units’ as defined in this paper. The interface between bone and dental follicle soft tissue is critical for tooth eruption, as it is only at this surface that bone is either deposited by osteoblasts as fresh osteoid, or alternatively resorbed by osteoclasts. Finite elements in soft tissue follicle are illustrated under differing levels of either tension or compression, marked with increasing intensities of green or red colour respectively. Soluble factors driving either bone formation (green arrows) or bone resorption (red arrows) are indicated as produced by cells residing in volumes described by the finite elements shown, such that where ‘green arrows’ predominate, bone deposition would occur, with bone resorption occurring where there is a preponderance of ‘red arrows’ marking resorptive factors. Cell responses to most stimuli are dose dependent, while a necessary assumption in this work is that there is a linear relationship between compression or tension quantitated in terms of hydrostatic stress in the current paper, and the amount of bone resorptive or formative soluble factor produced by cells. Finite elements vary greatly in volume, so that the number of cells and hence total quantity of bone resorptive or stimulatory soluble factors must vary in direct proportion to finite element volume. To allow for variability in finite element volume and permit meaningful quantitation of the biological impact of compression and tension across finite elements, we have multiplied the volume by hydrostatic stress within individual finite elements, and thus defined a new measure we term the ‘Biological Response Unit’.

Our intent in defining BRU is illustrated in Figure 5. Soft tissue follicle cells residing in volumes described by discrete FEs would experience defined levels of tension or compression during bite force application. As indicated in our earlier work, the boundary between soft tissue and the adjacent bone is the critical interface for eruption associated bone remodelling, as this is where bone is either deposited by osteoblasts laying down osteoid, or alternatively the bony surface may be resorbed by stimulated osteoclasts [54]. Soluble factors released by soft tissue cells under tension or compression, diffuse through the soft tissues to instruct either bone formation or resorption respectively [23]–[27]. BRU provides a means whereby differing levels of hydrostatic stress across FEs are normalized relative to FE volume, and thus captures the dual concepts of dose response to increasing levels of hydrostatic stress, and the impact of differing numbers of biologically active cells in differing FE volumes.

BRU values were determined for each FE in apical and coronal soft tissue caps under both bite force conditions studied. Positive BRU values were then summated to determine the total biological response to tension, as opposed to negative BRU values representing the overall biological response to compression. These were then expressed as relative percentage values of the absolute value of total BRU, such that a primarily compressive biological response was concluded when the relative percentage of compressive BRU exceeded 50%, and tension was concluded as biologically predominant when the relative percentage of compressive BRU was less than 50%. Higher and lower relative percentage values for compressive BRU were assumed to correspond to increasingly significant biological responses to compression or tension respectively.

BRU data was visualized in graphs in a similar fashion to that described for hydrostatic stress data above, but plotting increasing ranges of BRU across not only the relative percentage of volume occupied, but also against the relative percentage of total summated BRU. Coronal BRU values were generally much higher than those for apical tissues, so that differing ranges of BRU were selected for graphical representation of the relative percentage distribution of absolute BRU values. Summated volumes and BRU values were calculated for all FEs in defined ranges of BRU, with rising increments of 0.00017 BRU between 0.00029 BRU and 0.00250 BRU in the case of coronal follicle soft tissues, and increments intervals of 0.000045 BRU between 0.000050 BRU and 0.000630 BRU in the case of apical follicle soft tissues. FE volumes above and below the maximum and minimum BRU values indicated were summated accordingly.

## Results

### Dental Follicle and Periodontal Ligament Displayed Greater Equivalent Strain than Bone

**Figure 6:**
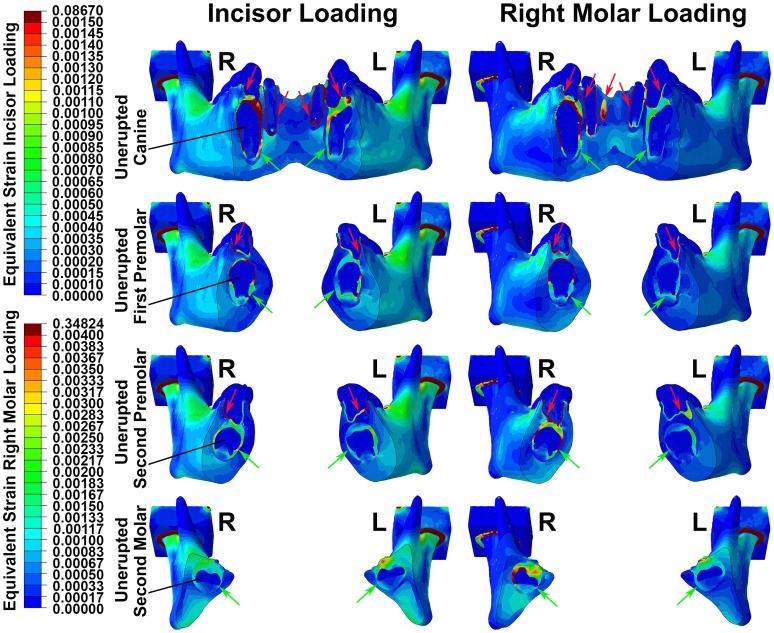
Colour plot diagrams showing patterns of equivalent strain. Four vertical sections of the mandible are shown, through each of the unerupted teeth during incisor loading (left side images) and right molar loading (right side images). Right (R) and left (L) joints are indicated respectively, while colour scales for incisor and molar loading images are shown separately. Irrespective of the pattern of loading applied, equivalent strain was maximal in soft tissues of the periodontal ligaments (red arrows) and dental follicles (green arrows), with generally lower levels of strain seen in hard tissues.

The highest equivalent strains induced by bite forces were in the soft tissues of the dental follicle and periodontal ligament, consistent with the lower stiffness of soft tissues relative to bone. This was the case for both erupted and unerupted teeth, as well as irrespective of whether incisive or unilateral molar bite forces were applied (Figure 6). Further, the effect was seen in teeth widely distant from the loaded incisors and molars respectively (Figure 6).

### A Greater Volume of Soft Tissue in Coronal Soft Tissue Caps of Unerupted Teeth Experienced Compression rather than Tension, and this was Reversed in Apical Soft Tissues where a Greater Volume Experienced Tension

**Figure 7:**
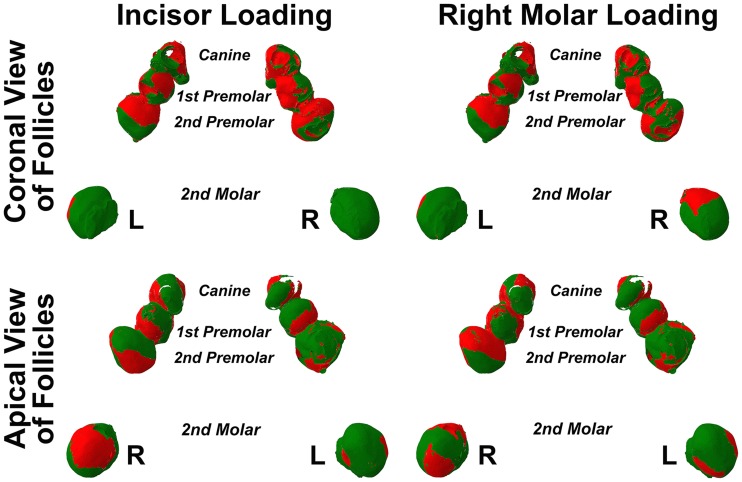
Dental follicle compression (red) and tension (green) during incisor or right molar bite force. The surface of dental follicles is seen from coronal or apical perspectives, while left (L) and right (R) sides are indicated. The upper surfaces of dental follicles for unerupted canines, first premolars and second premolars appeared subject to greater compression during both incisor and right molar loading, as compared with the lower surfaces of the same teeth which were in general subject to greater tension. This general pattern did not, however, appear to apply in the case of the unerupted second molars.

Examination of the lamina dura and bony crypts of both erupted and unerupted teeth failed to reveal any consistent pattern of strain to account for bone formation or resorption related to pre- and post-eruptive tooth movement. Examination of the soft tissue dental follicles of unerupted teeth, on the other hand, suggested broad zones of compression in follicle overlying crowns, with opposing broad zones of tension in follicle beneath root apices (Figure 7). Although this pattern was seen in the unerupted premolar and canine teeth, it seemed absent in the unerupted second molar teeth where tension was usually seen.

**Figure 8:**
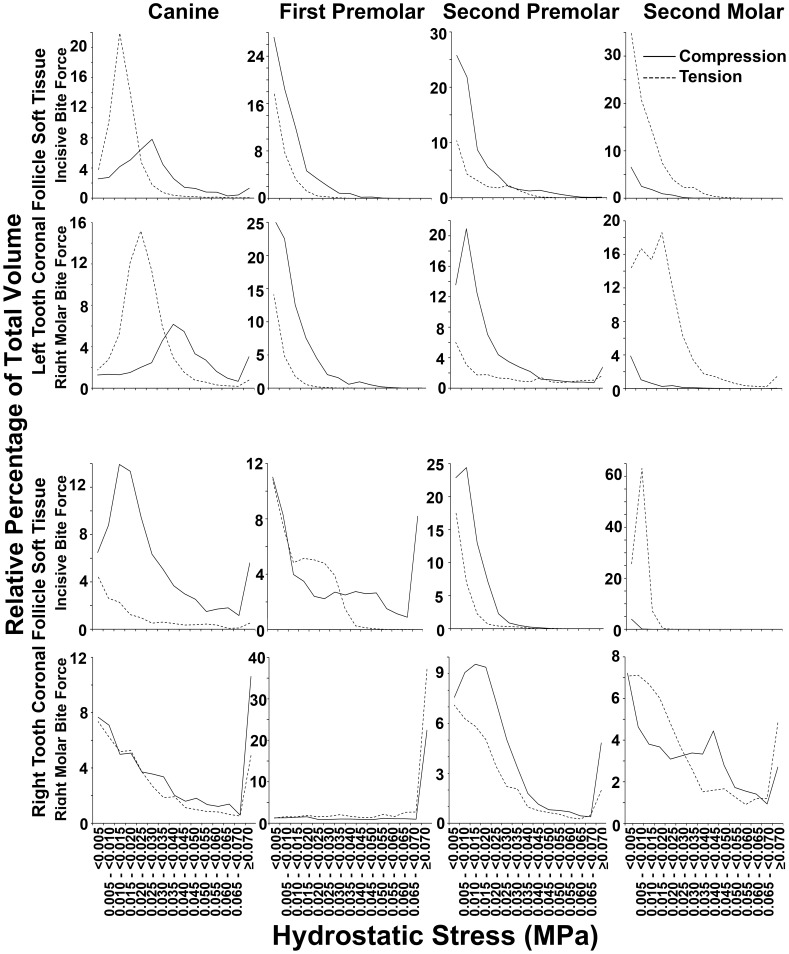
Percentage distribution of coronal soft tissue follicle volume according to the range of hydrostatic stress. Data for coronal soft tissue caps from each unerupted tooth is shown. For canines and premolars known to undergo active eruption at this stage of development, generally greater volumes had compressive (solid lines) as opposed to tensile (dashed lines) hydrostatic stress across most hydrostatic stress ranges. Tension, however, appeared more prominent in second molars which do not erupt at this stage of development. Further exceptions were in the left canine during incisive and molar bite force application, as well as in the right first premolar during right molar loading.

**Figure 9:**
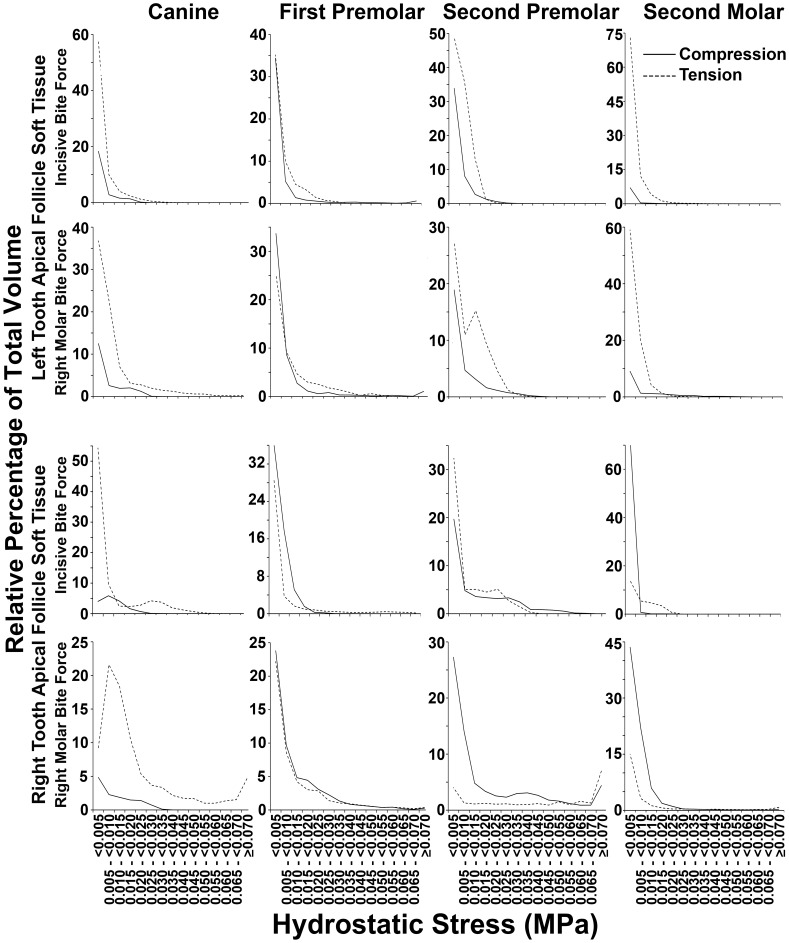
Percentage distribution of apical soft tissue follicle volume according to the range of hydrostatic stress. Data for apical soft tissue caps from each unerupted tooth is shown. Generally greater volumes had tensile (dashed lines) as opposed to compressive (solid lines) hydrostatic stress across most hydrostatic stress ranges. Exceptions to this pattern were seen, however, in the right first premolar and right second molar during incisive bite force application, as well as in the two first premolars and the right second premolar and molar, during right molar force application.

The relative percentage of tissue volume affected with compression as opposed to tension was determined for all soft tissue caps in unerupted teeth, and these data are shown in Table 2, as well as in greater detail in Figures 8 and 9. A proportionately greater volume of tissue experienced compression in coronal tissues as opposed to tension, and this was reversed in apical soft tissues. Particular exception was seen amongst the unerupting second molars, although there were also further exceptions in 7 out of a total of 24 potential instances of the canines and premolars (Table 2). Examining Figures 8 and 9, it was interesting to note often similar graphical profiles for incisive compared with right molar bite force loading within individual teeth on the left hand side. This graphical similarity seemed reduced on the right hand side, presumably because right molar loading would have distorting local effects.

**Table 2:** Relative percentage values for compression considering volume (Rel. % of Volume) and Biological Response Units (Rel. %BRU) during application of incisive or molar bite force in individual teeth.

	The Relative Percentage of Volume or BRU for Compression in Individual Teeth							
	Coronal Soft Tissue Follicle				Apical Soft Tissue Follicle			
	Canine	1st Premolar	2nd Premolar	2nd Molar	Canine	1st Premolar	2nd Premolar	2nd Molar
**Left Teeth Under Incisive** **Bite Force**								
Rel. % Volume	42.2 **#**	69.3	74.1	12.6 **#**	24.2	44.6	32.0	8.0
Rel. % BRU	57.9	79.1	73.8	9.6 **&**	22.4	45.2	25.0	5.6
**Left Teeth Under Right** **Molar Bite Force**								
Rel. % Volume	38.4 **#**	78.5	75.4	6.5 **#**	20.3	50.2 **#**	31.0	15.0
Rel. % BRU	51.0	89.0	67.8	2.9 **&**	14.5	46.7	24.4	26.8
**Right Teeth Under Incisive** **Bite Force**								
Rel. % Volume	84.5	56.1	71.4	4.1 **#**	16.4	61.0 **#**	43.5	72.0 **#**
Rel. % BRU	89.4	74.7	79.7	0.8 **&**	16.8	51.7 **&a**	54.0 **&a**	46.6
**Right Teeth Under Right** **Molar Bite Force**								
Rel. % Volume	56.0	37.9 **#**	62.1	47.9 **#**	12.7	52.8 **#**	73.8 **#**	76.4 **#**
Rel. % BRU	64.5	39.8 **&**	67.3	49.6 **&a**	6.5	52.4 **&a**	52.9 **&a**	64.5 **&**

With the exception of second molars, a general pattern of compression in coronal tissues and tension in apical tissues predominated. Considering the relative percentage of volume under compression in canines and premolars only, exceptions (#) were seen in 7 out of 24 instances. Further refinement by evaluation of BRU reduced exceptions to 5 out of 24 (&). 4 of these exceptions (&a) in BRU were within 4 percentage points of the 50% value marking consistency with the general rule, and 3 of these were in right sided apical tissues during right molar biting, likely representing localized asymmetrical effects of molar bite force.

**Figure 10:**
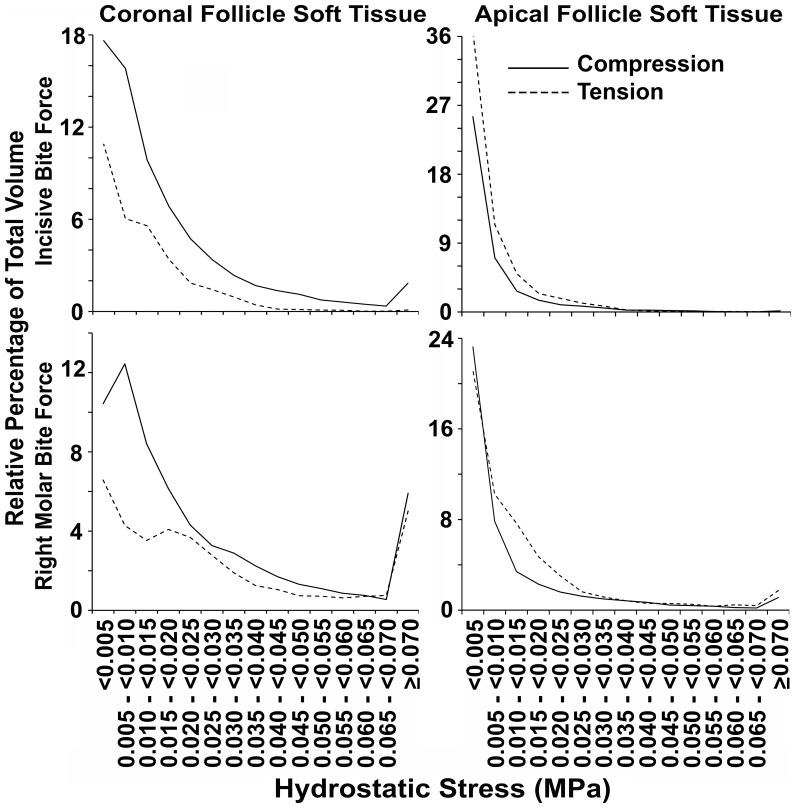
Percentage distribution of coronal and apical soft tissue follicle volume according to hydrostatic stress. Data for apical and coronal soft tissue caps, pooled separately from canines and premolars, under incisive or right molar bite force application are shown. For almost all bite force and hydrostatic stress conditions, greater volumes were devoted to compression (solid lines) in coronal follicle tissues, and to tension (dashed lines) in apical follicle tissues.

To examine general patterns in the absence of idiosyncratic local effects, data were pooled for canines and premolars (Table 3 and Figure 10), while the second molars were excluded from this pooled analysis on grounds that these teeth do not erupt at this stage of development.

**Table 3:** Relative percentage values for compression considering volume (Rel. % of Volume) and Biological Response Units (Rel. %BRU) during application of incisive or molar bite force in pooled incisors and premolars.

	The Relative Percentage of Volume or BRU for Compression in Pooled Canines and Premolars			
	Coronal Soft Tissue Follicle		Apical Soft Tissue Follicle	
	Incisive Bite Force	Right Molar Bite Force	Incisive Bite Force	Right Molar Bite Force
Rel. % Volume	68.8	62.3	40.1	44.9
Rel. % BRU	80.9	58.3	25.2	40.1

Compression in coronal tissues and tension in apical tissues predominated considering both volume and BRU separately, in both bite force conditions studied.

### The General Pattern of Predominant Compression in Coronal and Tension in Apical Follicle Soft Tissues of Unerupted Teeth was Strengthened when Analysis was of Biological Response Units

Tables 2 and 3 also show the relative percentage of compression in coronal and apical soft tissues as expressed in the absolute value of summated biological response units. When this more biologically relevant measure was applied, the second molars remained outside of the usual pattern seen, but the number of exceptions observed amongst canines and premolars was reduced to 5 out of 24 potential instances. Notably, all the 3 exceptions previously seen on the left hand side when volume alone was considered, were resolved when analysis was with BRU (Table 2). On the right hand side, the four exceptions noted from volume analysis remained, but three of these were substantially reduced to be within 3 percentage points of consistency with the general pattern, with only one of these instances not showing appreciable change but being nonetheless only 0.4% from the expected minimum 50% value. Nonetheless, BRU analysis did generate a single new exception in the apical right second premolar during incisive bite force.

**Figure 11:**
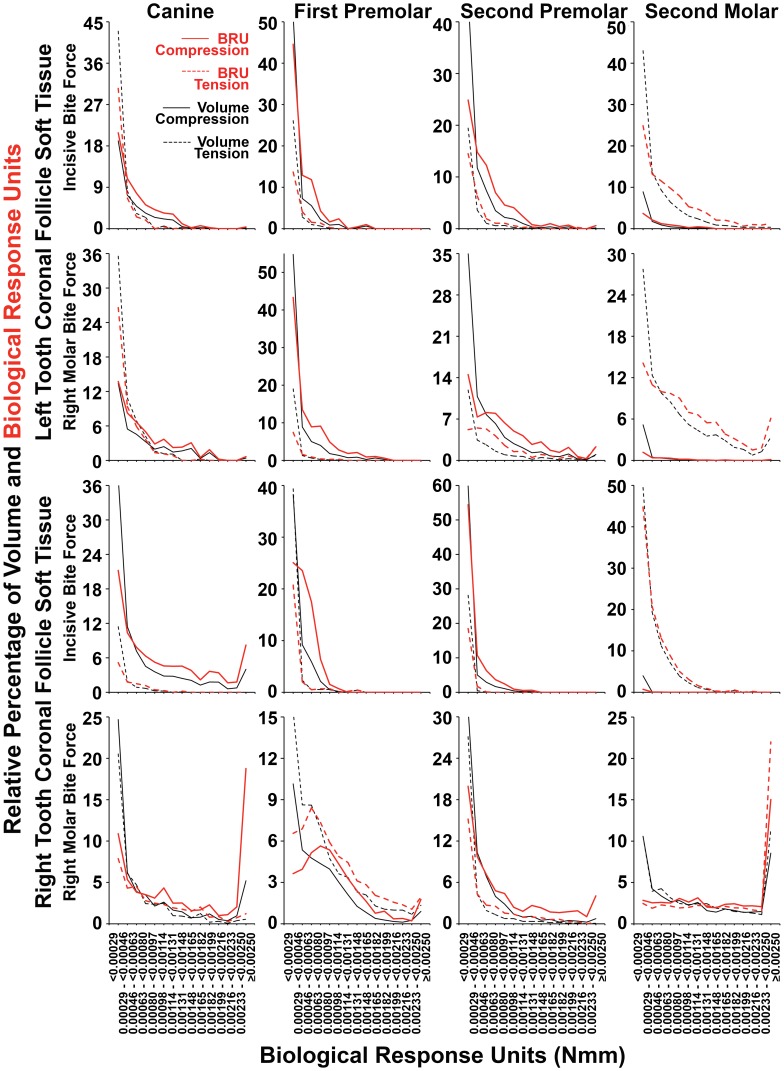
Percentage distribution of coronal follicle volume and summated BRU according BRU. As summarized in Table 2, considering tissue volumes alone (black lines), there was a strong tendency for compression (solid black lines) to dominate over tension (dashed black lines) across most BRU ranges. This was more pronounced when summated BRU was considered (red lines), such that there was a general right shift in BRU curves for compression (red solid lines) and sometimes a corresponding left shift in BRU curves for tension (dashed red lines). Exceptions for both volume and summated BRU were seen in the second molars, as well as in the right first premolar during right molar bite force application, while there were further exceptions considering volume alone in the left canine during both incisive and right molar loading.

**Figure 12:**
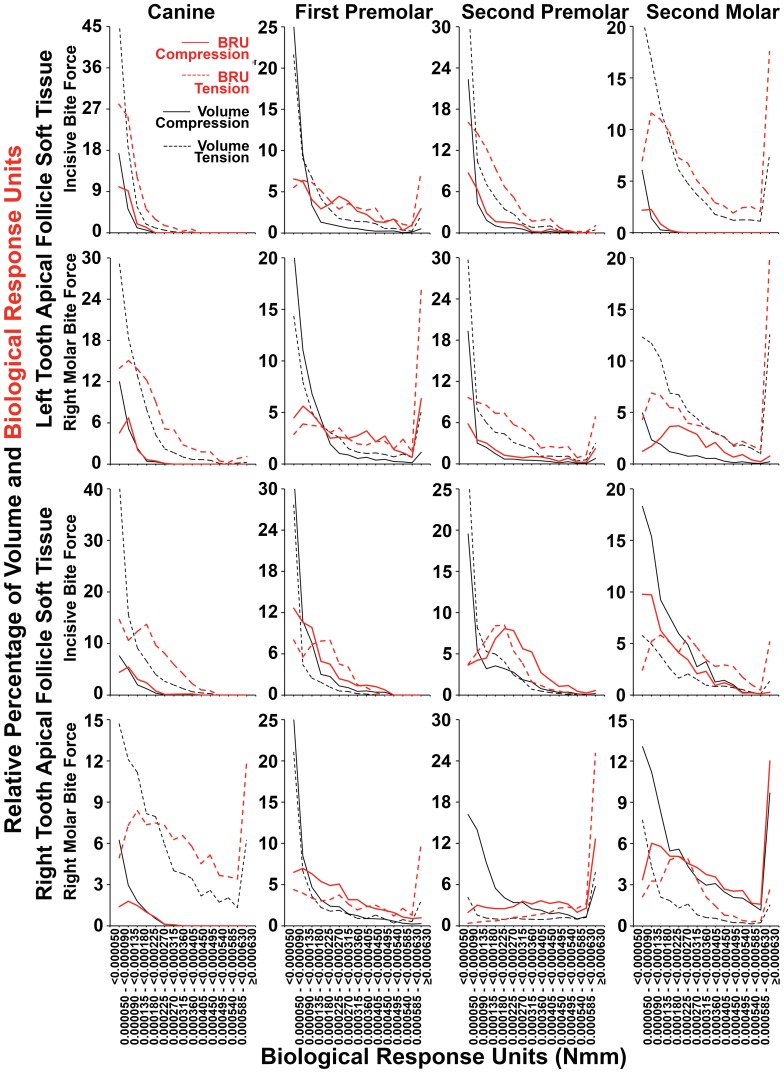
Percentage distribution of apical follicle volume and summated BRU according BRU. As summarized in Table 2, considering tissue volumes alone (black lines), there was a strong tendency for tension (dashed black lines) to dominate over compression (solid black lines) across most BRU ranges. This was more pronounced when summated BRU was considered (red lines), such that there was a general right shift in BRU curves for tension (dashed solid lines) and occasionally a corresponding left shift in BRU curves for compression (solid red lines). Exceptions occurred during right molar bite force with regard to both volume and BRU in the right premolars and second molar, and in the right first premolar during incisive bite force. Compression was also more dominant in incisive bite force application considering volume alone in the right second molar, and BRU alone in the right second premolar. Similarly, compression was only slightly dominant considering volume alone in the left first premolar during right molar loading.

**Figure 13:**
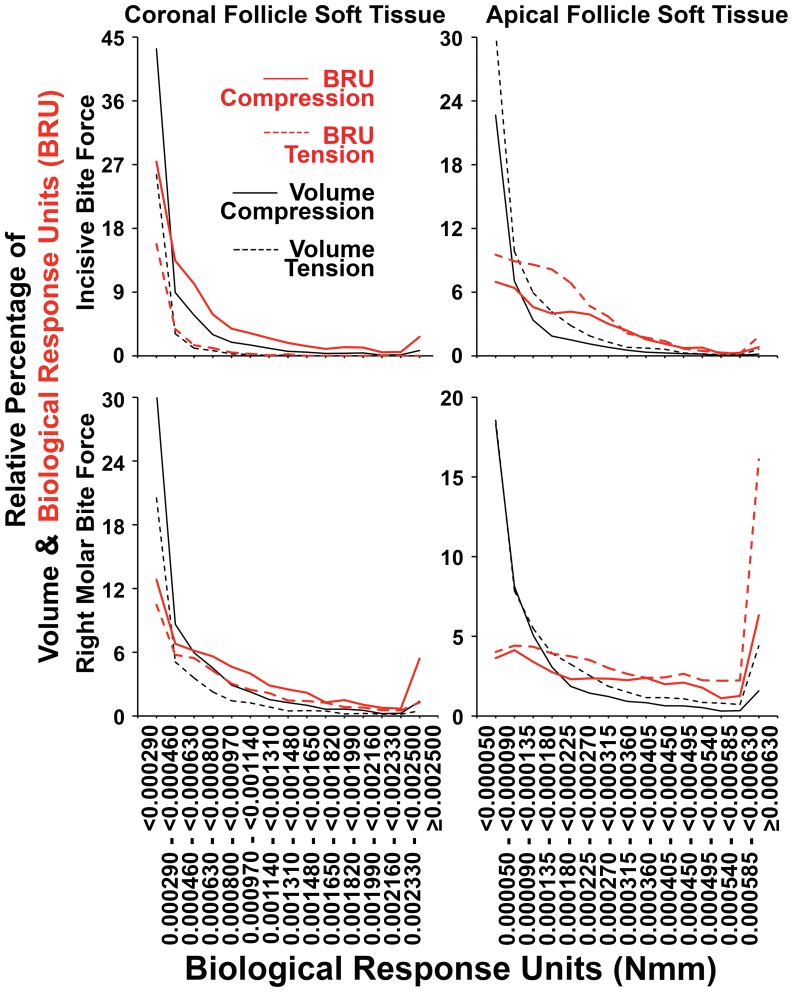
Pooled canine and premolar percentage distributions of follicle volume and summated BRU according BRU. As summarized in Table 3, considering tissue volumes alone (black lines), compression (solid black lines) dominated over tension (dashed black lines) in coronal tissues, and this was reversed in apical tissues. These patterns were more pronounced when summated BRU was considered (red lines), such that there was a general right shift in BRU curves for compression in coronal tissues (solid red lines), and for tension in apical soft tissues (dashed red lines), which a less pronounced right shifting of BRU tension and compression curves in coronal and apical tissues respectively.

Figures 11 and 12 show the relative percentage distributions according to the range of the absolute value of BRU for both compression and tension, in terms of both tissue volume occupied and summated BRU. Graphs are consistent with the data shown in Table 2 as well as in Figures 8 and 9. Further, there was a strong tendency for the BRU curves to be similar within individual apical or coronal tissues, irrespective if incisive or right molar bite force was examined, while the most conspicuous exceptions were on the right hand side where right molar bite force application may account for local differences. As expected from first principles, relatively large soft tissue volumes with low BRU levels (black lines), contributed much less to total BRU (red lines) compared with FE having high BRU levels. The effect of this, was that there was a general right shift in the relative percentage BRU curves (red lines) for compression and tension compared with volume (black lines). The effect of this right shifting, was to bring patterns of compression and tension more into alignment with the general expectation that there would be compression in coronal tissues, and tension in apical tissues. Figure 13 shows similar results for pooled data from canines and premolars, consistent with the general concept that bite force generates compression in coronal soft tissue follicles, and tension in apical soft tissue follicles.

## Discussion

We suggest that pre-eruptive tooth movement is a result of bone remodelling on the inner surface of the bony crypt and lamina dura, and that this is driven by strain and hydrostatic stress sensed by the soft tissues in intimate contact with this critical bony surface. Orthodontic tooth movement is premised on resorption of the bony surface of the lamina dura where there is compression, coupled with bone deposition under tension [27], [81]. Remodelling of separate bony surfaces is similarly correlated with compression and tension [61], [62], so that our observation of overall compression covering crowns of erupting teeth, as well as of overall tension in soft tissues at apical sites, is consistent with our suggested mechanism for tooth eruption.

There was marked visual similarity in the relative percentage hydrostatic stress volume and BRU graphs for individual teeth, irrespective if incisive or right molar bite force was studied, indicating a strong effect of local anatomical idiosyncracity on the results from individual teeth. We note that for bite forces to have a consistent general eruptive effect, that the pattern of response in individual teeth would need to be relatively independent of where the force is applied in the mouth, so that similarity of data across bite force conditions supports our suggested role for functional forces in tooth eruption. Exception to this general similarity was most apparent in right hand teeth during right molar loading, however, it seems reasonable to account for this on the basis of highly localized asymmetric effects.

Consistent with this interpretation, was that the most prominent exceptions to the general pattern of coronal compression and apical tension, were also in right hand teeth during right molar loading. While at first sight, this does seem to undermine universality of our suggested mechanism for eruption, it must be noted that normal chewing involves incisive as well as separate episodes of left and right molar loading. In consequence, notwithstanding episodes of reversed compressive and tensile loading of follicle during ipsilateral molar biting, combined incisive and contralateral molar loading would result in predominant patterns of hydrostatic stress consistent with our model.

Progressive refinement of analysis from simple volumetric determination of compression and tension, to considering the BRU reflective of biologically characteristic dose effects, was increasingly consistent with the model for tooth eruption proposed, and this also increases confidence in our analysis.

Further, because functional stress resulted in greater strain in dental follicle and periodontal ligament than in bone, these soft tissues seem ideally placed to act as relevant stress sensors. For sensor function to have biological effect, a means of coupling sensor to effector must be invoked, and the localization by others of soft tissue cells expressing bone resorptive and stimulatory biological activities [24], [82]–[84], is consistent with our hypothesis and data. Also supporting the idea that dental follicle and periodontal ligament soft tissues are the critical sensor organs for pre- and post-eruptive tooth movement, is that both bone resorption by osteoclasts and bone deposition by osteoblasts involved with tooth movement, are critical surface phenomena at the interface between soft and bony tissues surrounding the developing tooth [54]. For these reasons, we argue that the soft tissues likely play a leading mechanosensor role in tooth eruption relative to bone.

Relevant to the current study and supporting a critical role for the dental follicle and its interaction with surrounding bony crypt in intraosseous tooth eruption, is that teeth lacking dental follicle do not erupt [85]. Further consistent with our findings, is that when unerupted teeth are experimentally replaced with metal or silicone replicas, eruption occurs comparable to that seen in normal teeth [86].

While teeth normally drift forward and upwards in the jaws, this does not occur in either osseo-integrated implants or when there is pathological fusion of the bone to teeth [87], [88]. This provides clinical confirmation of the importance of the soft tissues in mediating tooth movement, further supporting the proposed model. Related to this, is that the periodontal ligament is recognized as having mechanosensor activity during orthodontic treatment [89]–[92].

The current study revealed a primarily tensile response in the soft tissues surrounding the unerupted second molars. While this might at first sight seem inconsistent with our conclusions, we note that at this stage of development, the molars are not erupting, so that our data for the second molars are confirmatory rather than in opposition to our model. Further, since tension is proposed as resulting in bone deposition, the generally tensile responses seen would have the effect of further entombing the second molar teeth during the pre-eruptive phase. We suggest that root formation combined with jaw growth would produce compressive coronal and tensile apical hydrostatic stress in the developing second molars, and that this would then result in eruption of these teeth at the appropriate developmental stage.

There was a single instance where BRU analysis revealed an inconsistently greater level of compression in the apical soft tissues of the right second premolar where analysis of volume was more consistent with expectation. This and remaining minor inconsistencies may reflect limitations inherent in our FE model, as vascular collapse and fluid displacement would permit some degree of reduced tissue volume upon compression in normal soft tissues, and the absence of comparable reduced volume effects in our model system may have slightly altered results. Measurements of mechanical properties in biological materials vary amongst studies and specimens and this can also influence the specific outcome of FE analysis [93], [94]. Nonetheless, the broadly similar outcome of incisive and right molar force application in the current study, suggests our FEA model to be sufficiently robust for meaningful interpretation of results.

It is also possible that molar biting generates more complex patterns of strain which fluctuate during different stages of individual bite-strokes, so that further analysis of hydrostatic stress patterns over time may be required to determine the role or otherwise of molar bite force in the mechanism we propose for directing bone remodelling. While the current study strongly supports our suggested new model for tooth eruption, we accept that further confirmation is required by similar analysis of both mandibles and maxillae from individuals at differing stages of development including the infant jaw before deciduous tooth eruption, as well as experimental animal studies.
